# Energetics contribution during no-gi Brazilian jiu jitsu sparring and its association with regional body composition

**DOI:** 10.1371/journal.pone.0259027

**Published:** 2021-11-12

**Authors:** Dalton Müller Pessôa Filho, Andrei Sancassani, Leandro Oliveira da Cruz Siqueira, Danilo Alexandre Massini, Luiz Gustavo Almeida Santos, Cassiano Merussi Neiva, Fred J. DiMenna

**Affiliations:** 1 Institute of Bioscience, Graduate Program in Human Development and Technology, São Paulo State University (UNESP), Rio Claro, Brazil; 2 Department of Physical Education, São Paulo State University (UNESP), Bauru, Brazil; 3 Division of Endocrinology, Diabetes and Bone, Department of Medicine, Icahn School of Medicine at Mount Sinai, New York, NY, United States of America; Poznan University of Physical Education, POLAND

## Abstract

We used measurements of metabolic perturbation obtained after sparring to estimate energetics contribution during no-gi Brazilian jiu-jitsu. Ten advanced grapplers performed two six-minute sparring bouts separated by 24 hours. Kinetics of recovery rate of oxygen uptake was modelled and post-combat-sparring blood-lactate concentration measured to estimate oxygen equivalents for phospholytic and glycolytic components of anaerobic energetics, respectively. Linear regression was used to estimate end-combat-sparring rate of oxygen uptake. Regional and whole-body composition were assessed using dual X-ray absorptiometry with associations between these measurements and energy turnover explored using Pearson’s correlation coefficient (significance, *P* < 0.05). Estimated oxygen equivalents for phospholytic and glycolytic contributions to anaerobic metabolism were 16.9 ± 8.4 (~28%) and 44.6 ± 13.5 (~72%) mL∙kg^-1^, respectively. Estimated end-exercise rate of oxygen uptake was 44.2 ± 7.0 mL∙kg^-1^∙min^-1^. Trunk lean mass was positively correlated with both total anaerobic and glycolytic-specific energetics (total, *R* = 0.645, *p* = 0.044; glycolytic, *R* = 0.692, *p* = 0.027) and negatively correlated with end-exercise rate of oxygen uptake (*R* = -0.650, *p* = 0.042). There were no correlations for any measurement of body composition and phospholytic-specific energetics. Six minutes of no-gi Brazilian jiu-jitsu sparring involves high relative contribution from the glycolytic component to total anaerobic energy provision and the link between this energetics profile and trunk lean mass is consistent with the predominance of ground-based combat that is unique for this combat sport. Training programs for Brazilian jiu-jitsu practitioners should be designed with consideration given to these specific energetics characteristics.

## Introduction

The popularity of the combat sport Brazilian jiu jitsu (BJJ) has increased in recent years [1, 2]. However, one drawback with such rapid growth is that sufficient research to inform BJJ training is presently lacking [1, 3]. Regardless of sport, athletic performance is multi-factorial with requirements unique to the sport. Consequently, a training program for an athlete should be highly specific and structured to hone physiological, biomechanical and anthropometric characteristics that contribute to optimizing performance in that particular sport [4]. With respect to physiology, components of specificity to address include energy system(s) contributing, muscle groups involved and performance skills required [5]. Research-based precision is necessary for determining a specific sport’s characteristics for each of these [5].

During BJJ competition, the objective is to take the individual with whom the grappler is competing to the ground and employ combat techniques to achieve positional control and, ultimately, submission via chokehold or joint lock [6]. Consequently, as opposed to standing combat, most of match play is ground based with both static and dynamic activation of the upper- and/or lower-body musculature performed with alternating periods of low- and high-intensity effort (e.g., at an ~8:1 ratio with the latter lasting ~2–5 s) [7]. Match duration spans 5–10 continuous minutes based on graduation level of the athlete and multiple matches are typically performed during each competitive event as winners advance.

A significant contribution of anaerobic metabolism to energy turnover during BJJ combat has been reported. For example, a number of studies have reported blood-lactate concentration ([lactate]_b_) exceeding 10 mmol∙L^-1^ immediately following a simulated match [6, 8, 9] with even higher values (e.g., 14.8 ± 3.2 mmol∙L^-1^) following actual competitive matches in international events [3]. However, post-combat elevation does not appear to differ based on graduation level of athlete [6] or whether the combat occurs in “gi,” the traditional uniform comprising loose-fitting pants and a jacket closed with cloth belt (i.e., gi vs. no-gi competition) [9]. The latter finding is interesting because “no-gi competition” tends to result in faster combat and an increased challenge of gaining control over the individual with whom the grappler is competing. Consequently, greater post-combat [lactate]_b_ elevation might be expected following this type of competition. Cross-sectional analysis of the fitness profiles of BJJ athletes also supports the importance of anaerobic energy transfer during combat. For example, Wingate-test values for peak and mean power (i.e., oft-used proxies for anaerobic capacity) for BJJ athletes are higher than those considered excellent for healthy people [1] whereas maximum rate of oxygen uptake ($\dot{V}O_{2\max}$; i.e., the universally recognized criterion measure of aerobic fitness) does not discriminate BJJ-performance capacity [1].

Considering the intensity/duration characteristics of muscle activation during BJJ combat [7] and the [lactate_b_] concentration that has been reported following it [3, 6, 8, 9], it stands to reason that BJJ training should be specifically designed to stress both phospholytic and glycolytic component of anaerobic metabolism. However, the degree to which training should be devoted specifically to each of these requires further clarification. This is important because while both of these forms of energy transfer fall under the “anaerobic” umbrella term, each is unique with respect to the training stimulus to which they optimally respond (e.g., when performing high-intensity interval training, specificity with regard to work interval intensity and duration, recovery interval intensity and duration, and work/recovery repetitions). Differentiating the relative contribution of the anaerobic components is also important for determining potential nutritional strategies and ergogenic aids that would be specific for optimizing performance given the requirements of a particular sport (e.g., strategies designed to increase the availability of phosphocreatine or decrease the acidification associated with anaerobic energy transfer). However, unlike aerobic contribution which can be directly measured by collecting gas exchange and ventilatory data during performance, anaerobic metabolism during athletic training/competition can only be estimated. One way to do so is by measuring metabolic values post exercise to predict the energetic event(s) that precipitated them [10, 11]. In addition to post-exercise measurement of the change in blood-lactate concentration post vs. pre combat, to quantify the glycolytic component during combat, oxidative recovery kinetics can be used to estimate the phospholytic and, in concert with the former, total anaerobic contribution [10, 12].

As previously mentioned, in addition to energy-system contribution, research is required to quantify muscle-group involvement in order to inform sport-specific training [5]. In this regard, much like athletes who participate in other combat sports, research confirms that BJJ athletes demonstrate a “mesomorphic” somatotype characterized by a high level of muscular development and low level of accumulated fat [1]. Moreover, Báez et al. [13] found that these anthropometric characteristics are associated with BJJ fighting style with guard-pass fighters demonstrating greater mesomorphy compared to fighters who rely more on the guard fighting technique. However, similar research defining how these anthropometric characteristics and, specifically, degree and location of lean mass are associated with energetic demands during BJJ combat is lacking. In addition to training specificity, this is important because like other combat sports, BJJ athletes in competition are classified according to body mass. Consequently, competitors have to make challenging decisions about “weight cutting” based on the balance between gaining an advantage by competing in a lighter weight class and the compromise in performance that can occur [14] and potential danger associated with excessive weight loss [15]. In this regard, in a recent paper, Barley et al. review current literature on the pre-competition weight-cutting methods that are often employed (e.g., energy-intake restriction, fluid reduction and prescription pharmaceutical misuse) in an attempt to define the threshold between acceptable weight cutting and that which impairs performance [16]. They conclude that it is presently not clear whether the benefit derived from qualifying to compete against a lighter individual via weight cutting offsets the potential reduction in performance that might occur on account of it [14] and, therefore, suggest that more research is required to develop a better understanding of how to safely and effectively manage weight when qualifying to compete in combat sports [16].

The purpose of this investigation was to use post-combat-sparring measurements of [lactate_b_] and oxygen uptake recovery kinetics to estimate the anaerobic energy provision (expressed as oxygen equivalents; O_2Eq_) for both phospholytic and glycolytic component during no-gi BJJ sparring. Consistent with what has been found for simulated matches involving other combat sports–for example, taekwondo [17], karate [18, 19] and judo [20]–and prescribed BJJ exercise “sets” performed discontinuously [21], we hypothesized that the energetics contribution from the phospholytic component during combat would be ≥50% of total anaerobic energy turnover. We also used dual-energy x-ray absorptiometry (DXA) to determine regional body composition for each participant so that the contribution of each anaerobic component could be “scaled” in accordance with anthropometric characteristics [22]. Given the degree to which the upper-body musculature is involved in BJJ sparring, we hypothesised that total anaerobic energy turnover during BJJ sparring would be associated with trunk and upper-limb regional and lean mass of the participant.

## Materials and methods

### Participants and ethical approval

Ten healthy male BJJ athletes (age, 24.7 ± 6.0 years; range, 20–40 years; stature, 178.5 ± 7.0 cm; body mass, 74.8 ± 7.9 kg) with expertise in the no-gi fighting modality participated in this investigation. Eligibility was determined based on age (≥18 years), sex (male), graduation level (purple, brown or black belt in BJJ), level of experience in competition/training (≥2 years in regional amateur competitions) and convenience insofar as the athlete’s availability to participate during the testing period. We also restricted inclusion to athletes within a similar weight range (67–83 kg) so that athletes within proximity in this regard could be matched. The investigation’s methodology, which conformed to the ethical principles of the Declaration of Helsinki, was approved by the São Paulo State University Ethical Committee (process: 016375 FC/UNESP). Participants were required to give written informed consent prior to beginning the study once experimental procedures, associated risks and potential benefits of participation had been explained.

### Sparring

Participants performed no-gi BJJ combat sparring for six minutes two times with 24 hours of rest interspersed. The BJJ athletes were instructed to arrive for testing in a hydrated state ~180 min after consuming lunch; however, no standardized meals were specified. The sparring took place in a room designated for combat-sport learning and training at São Paulo State University on a standard 10 x 10 meter BJJ tatami mat. We chose six-minute duration to ensure that combat-sparring duration was appropriate for all of our participants based on the fact that BJJ matches are typically five minutes long for individuals with white belts, 6–8 minutes long for more advanced belts and 10 minutes long for black belts. We also reasoned that six minutes of sparring provided an adequate period of time to elicit a high degree of both anaerobic and aerobic energy turnover. If submission occurred, fighters restarted combat sparring immediately in the “za-rei” start position (the traditional Japanese bow from the kneeling position). A free-style technique was employed by the participants with no-gi BJJ rules enforced. Specifically, the technique involved one-on-one sparring where partners resisted and countered each other’s moves in a continuous “flowing” manner thereby testing both attack (joint manipulation, immobilisation, asphyxiation) and defense (scaping, sweeping, passing) with no planned sequence. In addition to body mass (see above), partners were selected based on graduation level with pairings remaining the same for both fights.

### Measurements

Prior to and following each 6-min session of sparring, pulmonary gas exchange and ventilation were measured continuously using a portable gas-analysis system (K4b^2^, Cosmed, Rome, Italy). Specifically, for 10 minutes prior to combat-sparring initiation and for seven minutes following its cessation, participants wore a respiratory mask so that inspired and expired gas volume and gas concentration signals could be continuously sampled. During these measurement periods, participants were instructed to remain seated. Before each test, the measurement unit was calibrated according to manufacturer recommendations. During combat sparring, the mask was removed; however, to prevent the delay that would occur when post-combat-sparring measurement resumed (≤15 s after cessation of combat sparring), airflow into it was maintained. Data derived during this period was identified and removed prior to analysis. Breath-by-breath values for the rate of oxygen uptake ($\dot{V}O_{2}$) for pre- and post-combat-sparring periods of each test were then exported for further analysis (see below).

Immediately prior to and at various time points following (specifically, at 60, 180, 300 and 420 s) combat sparring, 25 μl of arterialized capillary blood was collected from the earlobe of each participant and stored in a microtube with 50 μl of NaF 1% solution. These whole blood samples were subsequently analyzed to determine [lactate_b_] using the electroenzyme method by an analyzer that was automatically calibrated prior to each analysis (YSI 2700, Yellow Springs, USA). For pre-combat-sparring measurement, one sample was taken upon completion of the 10-min period of resting data collection whereas post-combat-sparring measurement comprised samples at 60, 180, 300 and 420 s following cessation. The two values recorded at each time point for the two sessions were averaged after which the highest value observed across the four post-combat-sparring values ([lactate_b_]_peak_) and the single averaged value obtained during the pre-combat-sparring measurement ([lactate_b_]_pre_) were used to calculate [lactate_b_] accumulation (i.e., the difference between these two values; [lactate_b_]_Δ_) for each participant.

Regional and whole-body mass, fat mass and lean mass excluding bone were measured by an experienced technician using dual-energy DXA (Hologic QDR Discovery Wi, MA, USA) according to manufacturer recommendations. For these measurements, participants wore light clothing and no shoes. We also ensured that participants had no metallic objects attached to either their body or clothes during these measurements. The participant was positioned supine on a flat table with their feet close together and arms placed parallel to their trunk. Lines were adjusted and aligned by the same experienced technician by identifying specific anatomical points as indicated by the unit software.

### Data analysis

Breath-by-breath $\dot{V}O_{2}$ data collected during the two pre-combat-sparring periods for each participant were averaged to provide a single pre-combat-sparring value ($\dot{V}O_{2pre}$). Breath-by-breath $\dot{V}O_{2}$ data collected following combat sparring were initially examined to exclude errant breaths caused by coughing, swallowing, sighing, etc., and those values lying more than three standard deviations from the local mean were considered for removal [23]. The remaining values were then linearly interpolated to provide second-by-second values that were time aligned to the start of data collection and ensemble averaged across the two post-combat-sparring measurements in order to improve signal-to-noise ratio. The first 15 s of data of the averaged post-combat-sparring measurements were then removed to eliminate the influence of the “cardiodynamic” phase [23] after which a nonlinear least-square algorithm was used to fit the data. Specifically, a biexponential model was employed to characterize the response into primary (“fast”) and slow components as per the following equation:

$$\dot{V}O_{2}(t)=\dot{V}O_{2\mathrm{post}}-A_{p}\times [1-e^{‐(t-{\mathrm{TD}}_{p})/\tau_{p}}]+A_{s}[1-e^{‐(t-{\mathrm{TD}}_{s})/\tau_{s}}]$$

where $\dot{V}O_{2}(t)$ represents the absolute $\dot{V}O_{2}$ at given time (*t*); $\dot{V}O_{2post}$ is calculated as the three-breath average $\dot{V}O_{2}$ at the onset of recovery; A_p_, TD_p_ and τ_p_ represent the amplitude, time delay and time constant, respectively, describing the primary decrease in $\dot{V}O_{2}$ during the recovery period; and A_s_, TD_s_ and τ_s_ represent the amplitude, time delay and time constant, respectively, describing the slow phase of the decrease.

In addition to the biexponential model that we used to quantify the time course of $\dot{V}O_{2}$ recovery kinetics, we also fit the initial 20 s of $\dot{V}O_{2}$ data following the 15-s exclusion for the cardiodynamic phase during recovery with a linear function as per the following equation:

$$\dot{V}O_{2}(t)=mx+b$$

where $\dot{V}O_{2}(t)$ represents the absolute $\dot{V}O_{2}$ at given time (*t*) and m and b represent the slope and intercept of the post-combat-sparring $\dot{V}O_{2}$ decline, respectively. These values were then used to estimate the $\dot{V}O_{2}$ at the end of combat sparring ($\dot{V}O_{2end‐est}$) via back extrapolation [24].

The phospholytic component of anaerobic energy turnover during combat sparring was calculated by estimating the “oxygen deficit” (O_2Def_) that was present at combat-sparring onset. Specifically, the O_2Eq_ for the phospholytic component was calculated as A_p_ × (TD_p_ + τ_p_) with subsequent adjustment for the reduction in the volume of O_2_ stores in the blood (assumed to be 550 mLO_2_ based on total blood volume of 5–6 L) that also comprises part of O_2Def_ [10]. The O_2Eq_ for the glycolytic component was calculated based on the assumption that the energy derived for each mM∙L^-1^ accumulation of [lactate_b_] is equivalent to the energy released by the consumption of 3.0 mLO_2_ per kg of body mass ([lactate_b_]_Δ_× 3) [10, 11].

### Statistical analyses

Data normality was checked by the Shapiro-Wilk test. The estimates for total anaerobic energy turnover, phospholytic v. glycolytic turnover and regional and whole-body composition measurements are presented as means ± standard deviations along with 95% confidence intervals of the estimates (95%CI) and standard errors of the mean (SEM). Correlations between: 1.) anaerobic energy turnover (both total and component specific) and regional/whole-body composition; and 2.) $\dot{V}O_{2end‐est}$ and regional/whole-body composition were assessed using Pearson’s correlation coefficient (*R*). Significance level was set at *p* < 0.05. To assess the strength of association for significant correlations, adjusted coefficients of determination (*R*^2^_adj_) were classified as small (≥0.04 to <0.25), medium (≥0.25 to <0.64) or strong (≥0.64) [25].

## Results

**Fig 1:**
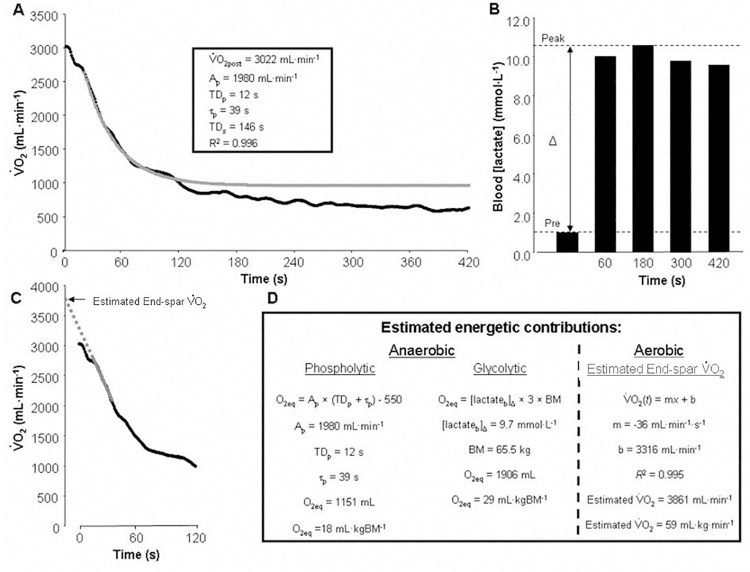
Graphical depiction of the metabolic data that were collected for a representative participant (age, 26 years; stature, 166 cm; BM, 65.5 kg; see participant number 8 in S1 Table for further information about this individual) immediately following BJJ combat sparring along with analyses that were performed to assess energetic characteristics during combat sparring. A six-minute session of combat sparring was done on each of two separate days so that like data could be averaged to increase signal-to-noise ratio. **Panel A** Second-by second $\dot{V}O_{2}$ data (expressed as black circles that form a line) with line of best fit for the primary phase of the decline, which was derived via biexponential modelling of seven minutes of post-combat-sparring data (grey line). The first 15 s of data were excluded from the fit to avoid contamination by the “cardiodynamic” response phase. Box inset lists the estimates revealed by the modelling procedure that were used to estimate the phospholytic contribution to energy turnover during combat sparring (calculations provided in **Panel D**). **Panel B** Blood [lactate] measurements obtained before (pre) and after combat sparring. Post-combat-sparring measurements were taken at four time points so that the peak value (upper vertical line) could be determined. The difference between this and the pre-combat-sparring value (lower dashed line) represents the peak blood [lactate] accumulation (Δ, vertical arrow) that was used to estimate the glycolytic contribution to energy turnover during combat sparring (calculations provided in **Panel D**). **Panel C** Second-by second $\dot{V}O_{2}$ data (expressed as black circles that form a line) with line of best fit for the initial decline, which was derived via linear modelling of the data collected from 15 to 35 s post combat sparring (i.e., with the first 15 s once again excluded to remove the cardiodynamic component) (grey solid line). Dashed grey line represents back extrapolation of the line of best fit to the point of combat-sparring cessation in order to estimate end-combat-sparring $\dot{V}O_{2}$ in an attempt to obtain information about the aerobic contribution to energy turnover during combat sparring (calculations provided in **Panel D**).

The group means ± SDs, 95%CIs and SEMs for the modelled estimates of the $\dot{V}O_{2}$ recovery kinetics are provided and data and analysis methods for a representative participant (age, 26 years; stature, 166 cm; BM, 65.5 kg) are depicted in Table 1 and Fig 1, respectively. The measured $\dot{V}O_{2pre}$ was 6.1 ± 1.3 mL∙kg^-1^∙min^-1^ (95%CI: 4.6–7.7 mL∙kg^-1^∙min^-1^; SEM = 0.4 mL∙kg^-1^∙min^-1^) and the estimated $\dot{V}O_{2end‐est}$ was 44.2 ± 7.0 mL∙kg^-1^∙min^-1^ (95%CI: 40.1–48.4 mL∙kg^-1^∙min^-1^; SEM = 2.2 mL∙kg^-1^∙min^-1^) based on a slope and intercept (*R*^2^ = 0.956 ± 0.047; 95%CI: 0.928–0.984; SEM = 0.015) of the $\dot{V}O_{2}$ decline at combat-sparring cessation of -25.7 ± 9.8 mL∙s^-1^ (95%CI: -31.4–-19.9 mL∙s^-1^; SEM = 3.1 mL∙s^-1^) and 2897 ± 360 mL∙min^-1^ (95%CI: 2686–3108 mL∙min^-1^; SEM = 211 mL∙min^-1^), respectively. Using the estimates for the primary phase of the $\dot{V}O_{2}$ off-kinetics response (Table 1), the O_2Eq_ estimate for the phospholytic component of anaerobic energy turnover during combat sparring was 1264 ± 636 mL (95%CI: 890–1638 mL; SEM = 201 mL). Stated relative to body mass, this value was 16.9 ± 8.4 mL∙kg^-1^ (95%CI: 12.0–21.8 mL∙kg^-1^; SEM = 2.7 mL∙kg^-1^).

**Table 1:** Estimates revealed by mathematical modelling of $\dot{V}O_{2}$ recovery kinetics following six minutes of no-gi BJJ combat sparring.

	Mean ± SD	95%CI	SEM
$\dot{V}O_{2post}$ (mL∙min^-1^)	2829 ± 320	2641–3017	101
A_p_ (mL∙min^-1^)	1811 ± 339	1611–2011	107
TD_p_ (s)	12 ± 4	10–14	1
τ_p_ (s)	49 ± 15	40–58	5
TD_s_ (s)	110 ± 42	85–134	13
*R* ^2^	0.990 ± 0.004	0.988–0.993	0.001

$\dot{V}O_{2post}$ = three-breath average $\dot{V}O_{2}$ at the onset of recovery; A_p_ = amplitude of the primary decrease in $\dot{V}O_{2}$ during the recovery period; TD_p_ = time delay of the primary decrease in $\dot{V}O_{2}$ during the recovery period; τ_p_ = time constant of the primary decrease in $\dot{V}O_{2}$ during the recovery period; TD_s_ = time delay of the slow decrease in $\dot{V}O_{2}$ during the recovery period.

The group means ± SDs, 95%CIs and SEMs for the pre- and post-combat-sparring values for [lactate_b_] are provided in Table 2. Of the four post-combat-sparring measurements, the peak value occurred at 60 s for two participants, 180 s for three participants, 300 s for four participants and 420 s for one participant. The [lactate_b_]_Δ_ was 14.9 ± 4.5 mmol∙L^-1^ (95%CI: 12.2–17.5 mmol∙L^-1^; SEM = 1.4 mmol∙L^-1^) resulting in an O_2Eq_ estimate for the glycolytic component of anaerobic energy turnover of 3398 ± 1312 mL (95%CI: 2627–4169 mL; SEM = 415 mL). The O_2Eq_ estimate for the total contribution of anaerobic energy turnover during combat sparring was, therefore, 4662 ± 1489 mL (95%CI: 3787–5537 mL; SEM = 471 mL). Stated relative to body mass, these values were 44.6 ± 13.5 mL∙kg^-1^ (95%CI: 36.6–52.5 mL∙kg^-1^; SEM = 4.3 mL∙kg^-1^) and 61.5 ± 14.9 mL∙kg^-1^ (95%CI: 52.8–70.2 mL∙kg^-1^; SEM = 4.7 mL∙kg^-1^), respectively. Proportionally, contributions to the anaerobic total were, therefore, 27.9 ± 11.9% (95%CI: 20.9–34.9%; SEM = 3.8%) and 72.1 ± 11.9% (95%CI: 65.1–79.1%; SEM = 3.8%) for the phospholytic and glycolytic components, respectively.

**Table 2:** Measurements of blood-lactate concentration prior to and following six minutes of no-gi BJJ combat sparring.

	Mean ± SD	95%CI	SEM
[lactate_b_]_pre_ (mmol∙L^-1^)	1.4 ± 0.4	1.1–1.6	0.1
[lactate_b_] at 60 s (mmol∙L^-1^)	14.2 ± 4.3	11.7–16.7	1.3
[lactate_b_] at 180 s (mmol∙L^-1^)	14.2 ± 4.5	11.5–16.9	1.2
[lactate_b_] at 300 s (mmol∙L^-1^)	14.8 ± 3.9	12.5–17.1	1.2
[lactate_b_] at 420 s (mmol∙L^-1^)	13.8 ± 4.1	11.4–16.2	1.3
[lactate_b_]_peak_ (mmol∙L^-1^)	16.2 ± 4.7	13.5–19.0	1.5

The group means ± SDs, 95%CIs and SEMs for the regional and whole-body composition measurements are provided in Table 3 and correlations between these values and the energetics variables we measured are provided in Table 4. Significant correlations were observed for both total and glycolytic turnover and trunk lean mass (total, *p* = 0.044; glycolytic, *p* = 0.027), upper-limb regional mass (total, *p* = 0.009; glycolytic, *p* = 0.017) and trunk regional mass (total, *p* = 0.002; glycolytic, *p* = 0.001). A significant correlation was also present for total and glycolytic turnover and lower-limb regional (total, *p* = 0.046; glycolytic, *p* = 0.034), but not lean (*p* > 0.05 in both cases) mass. There were no significant correlations for phospholytic turnover and any of these measurements of regional or whole-body composition (*p* > 0.05 in all cases; see Table 4). The $\dot{V}O_{2end‐est}$ was significantly negatively correlated with trunk lean mass (*p* = 0.042). With respect to the strength of the significant correlations that were observed (see above), trunk lean and regional mass exerted medium and strong effects on the variation in total anaerobic energy turnover, respectively. Trunk lean mass also exerted a medium (negative) effect on the variation in $\dot{V}O_{2end‐est}$.

**Table 3:** Regional and whole-body composition measurements for the 10 BJJ practitioners that participated in this investigation.

	Mean ± SD	95%CI	SEM
Lean mass			
Whole body (kg)	61.4 ± 5.3	58.3–64.5	1.7
Appendicular (kg)	30.7 ± 2.7	29.1–32.3	0.9
Body lean index (kg∙m^-2^)	20.2 ± 1.4	19.3–21.0	0.5
Upper limb (kg)	8.1 ± 0.8	7.6–8.5	0.3
Lower limb (kg)	21.1 ± 1.9	20.0–22.2	0.6
Trunk (kg)	28.8 ± 3.0	27.1–30.6	0.9
Total regional mass			
Upper limb (kg)	9.5 ± 1.0	8.9–10.1	0.3
Lower limb (kg)	25.9 ± 2.3	24.5–27.3	0.7
Trunk (kg)	34.1 ± 5.0	31.2–37.0	1.6

**Table 4:** Correlations between regional and whole-body measurements of body composition and measurements of energetic variables for the 10 BJJ athletes that participated in this investigation.

	$\dot{V}O_{2end‐est}$ (mL∙kg^-1^∙min^-1^)	Phospholytic (mLO_2_)	Glycolytic (mLO_2_)	Total Anaerobic (mLO_2_)
Lean Mass				
Whole body (kg)	-0.62*	0.05	0.60	0.56
Appendicular (kg)	-0.56	0.07	0.45	0.43
Body lean index (kg∙m^-2^)	0.05	0.31	0.49	0.58
Upper limb (kg)	-0.55	0.12	0.24	0.27
Lower limb (kg)	-0.47	0.03	0.46	0.43
Trunk (kg)	-0.65*	0.08	0.69*	0.65*
Total regional Mass				
Upper limb (kg)	-0.46	0.31	0.73*	0.77*
Lower limb (kg)	-0.46	0.07	0.67*	0.64*
Trunk (kg)	-0.39	0.20	0.87*	0.86*

* *p* < 0.05

## Discussion

The principal original finding of this investigation is that six minutes of no-gi BJJ combat sparring requires an ~72% contribution from the glycolytic component to total anaerobic energy turnover. This refutes our first hypothesis and likely reflects the marked differences between BJJ combat and other forms of combat sport that are predominantly based on striking/throwing with less/no ground combat. Conversely, our second hypothesis was supported as we found that the total anaerobic energy contribution during combat sparring is associated with trunk lean, upper-limb regional and trunk regional mass of the participant.

In this study, we measured $\dot{V}O_{2}$ kinetics and [lactate_b_] accumulation immediately following six minutes of no-gi BJJ sparring to estimate phospholytic and glycolytic contributions to anaerobic energy turnover during this form of combat [10, 11]. To the best of our knowledge, this is the first study to partition anaerobic energy turnover during BJJ combat sparring in this manner; however, similar measurements have been made for other combat sports. For example, Campos et al. [17] found that of the ~35% of total energy turnover provided by anaerobic metabolism during a simulated taekwondo competition (three two-minute rounds interspersed with one-minute recovery periods), the phospholytic component was responsible for ~85% of energetic provision. Similarly, during a series of four karate kumite fights, Beneke et al. [18] report ~23% anaerobic contribution that comprised ~65% reliance on phospholytic turnover. Our finding of ~72% glycolytic contribution to anaerobic energy turnover during BJJ sparring is, therefore, not consistent with this prior research. However, it is important to note that taekwondo and karate are combat modalities that only involve striking. Consequently, these sports require explosive muscle actions performed from a standing posture intermittently throughout a fight. This contrasts BJJ, which is predominantly ground based with relatively continuous combat. Considering these differences, a reduced phospholytic-to-glycolytic reliance might be expected for BJJ compared to these other combat sports. However, a phospholytic contribution of ~72% to the ~30% of total energy turnover provided by anaerobic metabolism has also been reported for judo-match simulation, a sport that involves ground-based combat that is similar to that which is employed in BJJ [20]. The reason(s) for this discrepancy compared to our findings is/are unclear, but might relate to the duration of combat that was investigated (1–5 min in the study of judo compared to 6 min for the present study), graduation level of athletes, the fact that we had competitors perform in accordance with a free-style “flowing” technique, the differences in temporal characteristics (e.g., effort time, pause time, effort/pause ratio) between the twop forms of combat and/or the fact that judo also involves a greater standing component resulting in increased intermittency of intense effort. Judo is also based on “throwing” the individual with whom the grappler is competing, an explosive action that would likely be more phospholytically based.

Another distinction that makes it difficult to reconcile our findings with previous ones for combat sports is that in addition to the contribution of the two anaerobic components, the intermittent, percussive nature of some combat sports allows for measurement of aerobic metabolism via collection of pulmonary gas-exchange/ventilatory data during combat [8]. Consequently, aerobic energy provision and, by extension, the proportionate involvement of each anaerobic component *in relation to overall energy turnover* can be determined based on the relative contribution of anaerobic energy turnover per se (i.e., as a percentage of a percentage). Conversely, combat sports that are predominantly based on grasping and clutching of the individual with whom the grappler is competing (e.g., BJJ, judo, wrestling) do not allow for such “real-time” direct measurement of aerobic metabolism without significantly restricting techniques being applied during analysis because wearing a ventilatory gas-collection mask during ecologically-valid simulation of performance is not feasible [8]. Consequently, for these types of sports, other than measuring gas exchange while participants performed a limited number of movements that typically comprise combat (e.g., see [20, 21] for judo and BJJ, respectively), the aerobic contribution during combat can only be estimated. We did so by using our post-combat-sparring measurement of gas-exchange/ventilatory data to estimate end-combat-sparring $\dot{V}O_{2}$ via back extrapolation [24]. While the single value so derived cannot be assumed to represent a “steady-state” provision of aerobic energy turnover during performance of activities like BJJ that involve oscillations in work rate, it is interesting to note that the value we observed (44.2 ± 7.0 mL∙kg^-1^∙min^-1^) was comparable to the peak reported during the performance of isolated 60-s BJJ sets involving takedowns and guard passes (43.9 ± 7.7 and 45.9 ± 8.4 mL∙kg^-1^∙min^-1^, respectively) [21]. With recognition of the aforementioned caveat, if values in this range do indeed approximate an average energetic requirement that was present throughout six minutes of combat sparring, aerobic metabolism would have been responsible for ~77% of total energy turnover with phospholytic and glycolytic components of anaerobic turnover providing ~6% and ~17% of the remaining requirement, respectively. However, it is important to note that this is merely speculation based on our post-combat-sparring $\dot{V}O_{2}$ measurement and associated estimation because, as previously mentioned, we could not directly measure $\dot{V}O_{2}$ while the participants were actually sparring. Consequently, we cannot state for certain regarding the proportional contribution of the oxidative system during this form of combat sparring.

Our findings regarding the high glycolytic contribution during BJJ sparring is consistent with the characteristics of BJJ being that this form of competition often comprises long periods of sustained grasping of the individual with whom the grappler is competing during ground-based combat. While intermittent explosive movements (i.e., ones that would be more reliant on contributions from the phospholytic branch of the anaerobic system of energy turnover) are also present during sparring, if/when the combat goes to “the ground,” exertion typically becomes more sustained. However, it is important to recognize that this would be influenced by the strategies being employed during a specific match. This caveat aside, in addition to skewing anaerobic metabolism toward glycolytic vs. phospholytic involvement [26, 27], sustained ground-based combat would also result in increased reliance on oxidative metabolism [28]. Unfortunately, as previously explained, the close body contact that is involved in this form of combat precludes the use of measurement devices that can be employed to collect gas-exchange data (and, by extension, quantify oxidative contribution) so we cannot draw any definite conclusions in this regard.

While the present study is, to the best of our knowledge, the first to quantify anaerobic provision during actual BJJ combat sparring in a component-specific manner, a number of previous ones have reported post-combat values for [lactate_b_] that are in line with what we observed. For example, Diaz-Lara et al. [3] measured [lactate_b_] for 26 expert male BJJ competitors immediately following competitive match play in the European Open Jiu-Jitsu Championship (match duration, 7–10 min) and found a maximal value of ~15 mmol·L^-1^ that was higher than what had been reported for simulated competition (e.g., 9–11 mmol·L^-1^). The authors attributed this difference to the higher level of athlete that would be encountered during international competition and/or the amplification of stress that can occur when performing in a high-pressure environment [3]. While the ~16 mmol·L^-1^ we observed for ([lactate]_b_)_peak_ following six minutes of sparring would not have been precipitated by either of these factors, it is important to note that we had participants compete in the no-gi manner which involves combat in normal athletic wear as opposed to traditional uniform. This is important because this difference in clothing markedly affects techniques that can be employed. For example, competing in gi allows for grasping of the collar, lapel, pants and sleeves of the individual with whom the grappler is competing to gain control and, ultimately, achieve submission. With this clothing-related assist removed during no-gi combat, competitors must rely more on body positioning and application of more advanced offensive techniques. Generally speaking, removal of gi gripping and the friction created by coverage of the skin also results in no-gi competition being faster and more action based. These factors would be predicted to increase energy expenditure per se and, by extension, glycolytic contribution that might be reflected in elevation in [lactate_b_] like the value we observed. However, Coswig et al. [9] compared gi and no-gi combat and, despite less total time of pause during the latter, found similar [lactate_b_] immediately following (peak, ~12 mmol·L^-1^), and five and 10 minutes into recovery after, a 10-minute no-gi simulated match. While the reason(s) for the greater elevation we found for no-gi combat sparring is/are unclear, one factor that might contribute was possible alteration of match strategy that would be expected during six compared to 10 minutes of combat. Regardless of these distinctions, however, given the growing popularity of no-gi competitions and the fact that BJJ athletes who compete in MMA fight without gi, more research is needed to clarify differences between this type of fighting and the traditional form and how those differences might affect sport-specific training for the BJJ and/or MMA athlete.

To provide additional information about energetic characteristics for no-gi BJJ combat sparring, we used allometric scaling to quantify the relationship between sources of energy turnover during combat sparring and regional mass measurements of the participant. This analysis revealed positive correlations for both total anaerobic energy turnover and regional mass in the trunk and upper limbs. We are aware of no research that has quantified energy provision based on body composition during performance of BJJ or other combat sports, but it is interesting to speculate that the greater upper-body involvement in ground-based combat compared to stand-up striking of other modalities and the reduced gripping in no-gi compared to gi BJJ performance each contributed to this finding. More definite is that phospholytic energy turnover was not correlated with any measurement of lean or regional body mass, which is consistent with the sustained combat actions that take place during match play. Finally, with the caveat regarding our inability to draw conclusions regarding aerobic energy turnover during combat recognized (see above), the fact that the positive correlation between trunk and upper-limb regional mass and anaerobic energy turnover is offset by a negative correlation between trunk lean mass and estimated end-exercise $\dot{V}O_{2}$ is consistent with the high-intensity nature of the upper-body pushing, pulling and squeezing that predominate during ground-based combat. It is also important to note that compared to the lower body, upper-body muscles typically comprise a greater proportion of type II fibers that rely more on anaerobic metabolism to support a given degree of effort [29]. Consequently, discounting technical expertise and match strategy employed during specific BJJ performance, our findings indicate that greater lean mass in the upper limbs and trunk are attributes that should predispose athletes to success in this sport based on the preponderance of sustained high-tension muscle activation (both dynamic and isometric) and burst concentric efforts by the upper-body musculature [30, 31].

With respect to training designed specifically for BJJ athletes, the implications of our findings are interesting to consider. Given the high-intensity intermittent nature of combat sports, high-intensity or all-out interval training (HIIT or SIT, respectively) have been suggested to be of great importance when designing training programs for such sports [32]. However, it is also well established that determining the characteristics of a HIIT sport-specific training regimen (e.g., work interval intensity and duration, recovery interval intensity and duration, work/recovery repetitions, etc.) should be based on the specific requirements of the sport. Our finding of a high glycolytic-to-phospholytic contribution to anaerobic energy turnover during no-gi BJJ combat sparring suggests that the “anaerobic” portion of a BJJ athlete’s training regimen should prioritize exercise that is specific for development of the “fast glycolytic system”; for example, 2–3 sessions per week comprising HIIT work intervals performed for 30 s at 90% of maximal work rate at a 1:2–1:3 work-to-recovery ratio [33]. Moreover, our findings regarding the relative importance of the trunk musculature with regard to establishing the high glycolytic energetic outlay during BJJ suggest that unlike conventional HIIT that is typically performed on a leg cycle ergometer, in addition to that type of training, a BJJ-specific program should also involve a modality that incorporates the upper-body musculature either exclusively (e.g., arm cranking) or in concert with the legs (e.g., rowing and/or exercise on an elliptical trainer) as part of the training regimen.

In addition to the inability to directly measure aerobic metabolism during BJJ performance, there are a number of limitations associated with the present study that require mention. Much like any combat sport that involves competing directly against an individual (as opposed to indirectly based on a scoring standard; for example, karate kata), the specific characteristics observed during a given BJJ match are highly dependent on the offensive and defensive techniques employed by the individual with whom the grappler is competing. Consequently, it is likely that the “average” energetic characteristics we have reported for BJJ combat sparring might vary considerably based on strategies employed, graduation level of athlete and, simply, circumstances with regard to how a match progresses. Any or all of these factors might have been responsible for the unusually-large discrepancies in [lactate_b_]_peak_ that we found across participants in this study (see S1 Table). Our results might have also been influenced by the age disparity of our participants (one of our 10 athletes was 40 years old while the other nine ranged from 20–29) and/or pre-competition meals that were ingested. It is also important to recognize that, as pointed out by Andreato et al. [34], while studies like ours provide information to distinguish between the physiological profiles of athletes of a specific combat modality (in this case, BJJ), such results should not be extended to MMA specifically because the physiological and metabolic demands during an MMA match are unique to requirements of that sport. Consequently, despite the fact that BJJ plays a preeminent role in the arsenal of many successful MMA athletes, our findings should not be applied to MMA training per se. Future research should, therefore, be designed to investigate the energetic demands that are specific for MMA combat as well as physiological and anthropometrical characteristics of competitors who are successful in that unique hybrid brand of combat sport.

## Conclusion

In conclusion, our post-combat-sparring analysis of the metabolic perturbation caused by six minutes of no-gi BJJ combat sparring indicates a high relative contribution from the glycolytic component to total anaerobic energy turnover. This contrasts other combat sports and likely reflects the fact that BJJ is predominantly ground based with minimal standing and throwing of the individual with whom the grappler is competing. The link between this energetic profile and trunk lean mass is also consistent with the unique aspects of combat that predominate during BJJ. More research is needed to determine the reproducibility of our findings and provide further information for formulating sport-specific training protocols for improving BJJ performance.

## Supporting information

S1 Table Raw data. (XLS)
